# The Antitumor Effect of Gene-Engineered Exosomes in the Treatment of Brain Metastasis of Breast Cancer

**DOI:** 10.3389/fonc.2020.01453

**Published:** 2020-07-30

**Authors:** Minchen Liu, Yulan Hu, Guiqian Chen

**Affiliations:** ^1^Engineering Research Center of Modern Preparation Technology of TCM, Innovation Research Institute of Traditional Chinese Medicine, Shanghai University of Traditional Chinese Medicine, Shanghai, China; ^2^Department of Cultural Heritage and Museology, Zhejiang University, Hangzhou, China; ^3^Zhejiang Provincial Key Laboratory of Silkworm Bioreactor and Biomedicine, College of Life Sciences and Medicine, Zhejiang Sci-Tech University, Hangzhou, China

**Keywords:** brain metastasis of breast cancer, CXCR4, TRAIL, exosome, MSCs, lentiviral vector

## Abstract

Strategies for treating brain metastases of breast cancer have demonstrated limited efficacy due to the blood–brain barrier (BBB). Gene therapy could improve the efficacy of chemotherapeutic drugs. Exosomes derived from the mesenchymal stem cells (MSCs) are small membrane-based gene vectors that can pass through the BBB. CXCR4 is the most commonly found chemokine receptor in human cancer cells. Furthermore, the SDF-1/CXCR4 axis plays an important role in the homing of MSCs for tumor cell diffusion and metastasis. TRAIL can selectively induce apoptosis in transformed cells without significant toxic side effects in normal tissues. In this study, exosomes were isolated from MSC^CXCR4+TRAIL^ transduced with CXCR4 and TRAIL using a lentiviral vector. Synergistic antitumor study showed that exosome^CXCR4+TRAIL^ exerted significant activity as a cooperative agent with carboplatin in an MDA-MB-231Br SCID mouse model, potentially engendering a novel strategy for advancing the treatment of brain metastases of breast cancer. Based on this study, further investigation of the effect of the vector on BBB and inducing apoptosis of brain tumors is warranted. In addition, the safety of the vector in animals during the treatment needs to be evaluated.

## Introduction

Brain metastasis has become the hot spot in the treatment of breast cancer due to its high morbidity, aggressiveness, and inaccessibility. Unfortunately, more than a quarter of breast cancer patients finally develop brain metastasis (1). Considering the high risk of surgery and the apparent neurotoxicity of brain radiotherapy, the use of chemotherapy, such as carboplatin, which is a first-line drug used in metastatic breast cancer therapy, is beneficial for treating orthotopic brain tumor, and brain metastases (2). However, the chemotherapeutic agents used for brain tumor treatment cannot pass across the blood–brain barrier (BBB) efficiently (3, 4). Thus, it is difficult to use chemotherapy to treat brain metastasis (5).

Nano-carriers have been developed to increase the ability of drugs to penetrate the BBB. Nevertheless, rapid drug clearance by the mononuclear phagocyte system (MPS) and toxicity of the nanoparticles have frequently been observed (6). Although polyethylene glycol is used to decrease drug uptake by the MPS, this can still result in reduced interaction among the target cells, which can decrease the drug distribution in the brain (7, 8). By comparison, exosome, a natural substance derived from the body, can penetrate into the BBB, which can improve drug transport to the brain by decreasing the influence of the MPS (9, 10). All of these characteristics make this system a promising therapeutic tool for treating brain diseases.

Exosomes are nano-sized carriers (30–120 nm) that are derived from different cell types such as B cells, T cells, epithelial cells, tumor cells, and mesenchymal stem cells (MSCs) (11–13). An ideal source of exosomes produces an abundance of exosomes that are clinically applicable. MSC is a unique human cell type, which has a capacity for large-scale preparation of exosomes (14, 15). MSCs also have characteristics that are beneficial for producing exosomes such as being easily accessible and non-immunogenic and having intrinsic therapeutic properties (14). Thus, MSC is the appropriate cell type for the large-scale production of exosomes.

Based on these considerations, we obtained the isolated and purified exosomes from MSCs transduced with lentiviral CXCR4 and lentiviral tumor necrosis factor-related apoptosis-inducing ligand (TRAIL, also known as Apo2L) (Exo^CXCR4+TRAIL^). CXCR4 and its ligand, SDF-1, are expressed extensively, including in immune cells, the brain, and the heart (16). Many studies have demonstrated that the SDF-1/CXCR4 axis plays an important role in processes including chemotaxis, stem cell homing, engraftment, expression of adhesion molecules, proliferation, and survival (17, 18). Thus, CXCR4 should lead to improved delivery of exosomes from MSCs to disease sites. Meanwhile, the molecule TRAIL, a member of the tumor necrosis factor ligand family, serves as a ligand for the four cellular membrane receptors, which is a death receptor activating the apoptotic program. As a type 2 transmembrane protein, TRAIL is expressed on the cell surface. It can be released by soluble TRAIL homotrimers. It has been observed that soluble TRAIL mostly causes apoptosis in tumor cells but not in normal tissues (19). In previous reports, the application of a combined medication for tumor cells with TRAIL and drugs has been evaluated for whether TRAIL could synergize with chemotherapeutic drugs (20–22).

The main principle of the scheme to deal with cancer treatment is that combination therapies can improve selectivity and efficacy in comparison to single-agent treatments. To explore the effect of combination CXCR4/TRAIL/carboplatin treatment for brain metastasis of breast cancer, this study examined the anti-tumor effect of Exo^CXCR4+TRAIL^ combined with carboplatin in an MDA-MB-231Br severe combined immunodeficiency (SCID) mouse model. Exo^CXCR4+TRAIL^ has been used as a cooperative agent with carboplatin against brain metastasis of breast cancer *in vivo*, providing the possibility of using this agent as a novel biological method for enhancing the treatment of brain metastases of breast cancer.

## Methods

### Materials

Carboplatin (C2538) was purchased from Sigma-Aldrich; Dulbecco's modified Eagle's medium (DMEM), fetal bovine serum, penicillin, trypsin, and streptomycin were purchased from Gibco BRL (Gaithersberg, MD, USA). All of the other chemical reagents used were of analytical grade.

### Animals

SCID mice and 3-week-old Sprague-Dawley (SD) male rats were supplied by the Experimental Animal Center, Zhejiang University, China. All of the animals were kept in 12-h light/dark cycle and (25 ± 1)°C temperature with free access to food and water. All animal procedures were performed in accordance with Health Guidelines for the Care and Use of Laboratory Animals of Zhejiang University, and the experiments were approved by the Animal Ethics Committee of Zhejiang University.

### Cell Lines

Brain-seeking human metastatic breast cancer cells were obtained from ATCC (USA). Cells stably transfected to express firefly luciferase (MDA-MB-231Br-Luc) were cultured in DMEM supplemented with 10% fetal bovine serum. Only cells from passages 2–10 were used. The human embryonic kidney 293T (293T) cells (GNHu17) were obtained from the Cell Bank of the Chinese Academy of Sciences (China) and were grown in DMEM containing 10% fetal bovine serum, 100 U/ml penicillin, and 100 mg/ml streptomycin. All of the cell lines were cultured at 37°C with 5% CO_2_.

### Isolation and Culture of MSCs

MSCs were isolated and cultured according to the protocol described in our previous report (23). MSCs were isolated from rat bone marrow cells obtained from the hind femurs of 3-week-old SD male rats. The cell suspension was placed into a 100-mm dish and cultured at 37°C in a humidified atmosphere containing 5% CO_2_. Sub-confluent cells from passages 2–6 were used for further study.

### Transfection, Virus Preparation, and Titer Determination

The CXCR4 gene was amplified using gene-specific primers containing the EcoRI and BamHI restriction sites, respectively. Polymerase chain reaction was performed, and the recombinant lentivirus vector was generated by cloning CXCR4 gene into the pLV-Puro vector as previously described (24). Then, 293T cells were transfected with the lentivirus vectors (CXCR4-GFP) in the presence of polybrene (8 μg/ml) for 13 h and switched to fresh medium. The titer of lentivirus vectors was (6.50 ± 0.5) × 10^8^ transforming units/ml. The supernatant containing the lentivirus containing CXCR4 and GFP (LV^CXCR4^) was collected, aliquoted, and stored at −80°C in a freezer. The lentivirus-containing TRAIL (LV^TRAIL^) plasmids were constructed as previously reported (25). Vector stocks were prepared according to published protocols (26, 27). Briefly, 1.8 × 10^7^ 293T cells in T150 flasks were transfected with 20 μg transfer vector, 14 μg helper plasmid, and 7 μg envelope plasmid by CaPO_4_ precipitation. The supernatant was collected after the transfection, filtered using 0.22 μm Stericups (Millipore, Bedford, MA), and centrifuged for 140 min at 20,000 rpm. The titer of lentivirus vectors was (7.25 ± 0.85) × 10^8^ transforming units/ml. The concentrated virus was dissolved in 100 μl phosphate buffer saline per 30 ml supernatant.

### Transduction of MSC by LV^CXCR4^

MSCs were cultured in six-well-plates at 5 × 10^5^ cells/well-concentration containing 4 μg/ml polybrene, and LV^CXCR4^ was added. The plates were centrifuged (1,200 rpm, 37°C, 1 h), and the medium was replaced by fresh medium after 6–16 h. The infected MSCs were then selected by continuous incubation with 10 μg/ml puromycin (Sigma) added, starting 1 day after transduction. Prior to further experiments, the MSCs overexpressing CXCR4 (MSC^CXCR4^) were cultured for 48 h without puromycin.

### Transduction of MSC ^CXCR4^ by LV^TRAIL^

MSC^CXCR4^ samples were cultured in six-well-plates at 5 × 10^5^ cells/well-containing 4 μg/ml polybrene, and LV^TRAIL^ was added. The plates were centrifuged (1,200 rpm, 37°C, 1 h). After 6–16 h, the medium was replaced by fresh medium.

### CXCR4 and TRAIL Expression in MSCs

The expression levels of CXCR4 and TRAIL in MSCs were further analyzed by Western blot. Briefly, MSCs and the MSCs overexpressing CXCR4 and TRAIL (MSC^CXCR4+TRAIL^) were lysed in a cell lysis buffer (Beyontime, China). Proteins of the two groups were separated by 10% sodium dodecyl sulfate-polyacrylamide gel electrophoresis. They were then transferred onto a nitrocellulose transfer membrane (Whatman). The membranes were first incubated with primary antibodies against CXCR4, TRAIL, and GAPDH (Abcam, Britain) and then with horseradish peroxidase labeled secondary antibodies. According to the manufacturer's instructions, the peroxidase activity was visualized with an enhanced chemiluminescence kit (Amersham Biosciences).

### Exosome Isolation

Exosome isolation from MSC^CXCR4+TRAIL^ and MSCs was carried out using total exosome isolation reagent (Invitrogen) as reported previously (28). The principal process was as follows: MSCs were cultured overnight and used when the confluence reached 80%. The MSCs were then washed with phosphate buffer saline and seeded in a hypoxic (1%) environment with serum-free media for 24 h. They were then centrifuged (Beckman Coulter Optima XPN-80, USA), and the supernatant was filtered through a 0.2-μm filter. About 100 ml of supernatant was concentrated to 500 μl using a membrane with a 100 kDa MWCO (Millipore). The concentrate and half the volume of total exosome isolation reagent were mixed well. The homogenous solution was centrifuged (4°C, 10,000 g, 1 h). The supernatant fluid was discarded, and the exosomes were resuspended in phosphate buffer saline. The isolated exosomes were stored at −80°C until use.

### Identification and Characterization of Exo^CXCR4+TRAIL^

Exo^CXCR4+TRAIL^, the exosome isolated from MSC^CXCR4+TRAIL^, was observed and photographed under transmission electron microscopy (TEM) (Tecnai G2 F20 S-Twin; FEI, Hillsboro, OR, USA) to get a better understanding of it. Furthermore, to identify the characteristics of exosomes, Western blot was carried out using the standard protocols described in section CXCR4 and TRAIL Expression in MSCs. The membranes were incubated with primary antibodies against CD63, CD81, CXCR4, ALIX, or Beta-Actin (BD Biosciences). They were further incubated with a chemiluminescent horseradish peroxidase substrate to detect primary antibody bound to the antigen present. In addition, the effect of CXCR4 and TRAIL on the characters of exosomes isolated from MSCs or MSC^CXCR4+TRAIL^ was identified by Western blot.

### Animal Tumor Model

MDA-MB-231Br-Luc cells (5 × 10^5^) were implanted intracranially into SCID mice as previously described (29). During the process, the appearance and behavior of the SCID mice were recorded daily, and no profound effects of distress and pain were observed. In order to evaluate the animal model, the brain of SCID mice was imaged with an *in vivo* imaging system on days 0, 14, 21, and 35 after cell implantation.

### Anti-tumor Study of Carboplatin Combined With Exo^CXCR4+TRAIL^ in Brain Tumors

Animal tumor models were grown for 14 days prior to carotid injection of carboplatin (5 mg/kg) combined with Exo^CXCR4+TRAIL^ (4 mg/kg) or exosome (4 mg/kg) on alternative days for 14 days. The mouse brain tumors in the two groups were then imaged with the *in vivo* imaging system.

### Statistical Analysis

The results were expressed as mean ± standard deviation. Statistical significance was set at *P* < 0.05.

## Results

### Identification of MSC^CXCR4+TRAIL^

**Figure 1:**
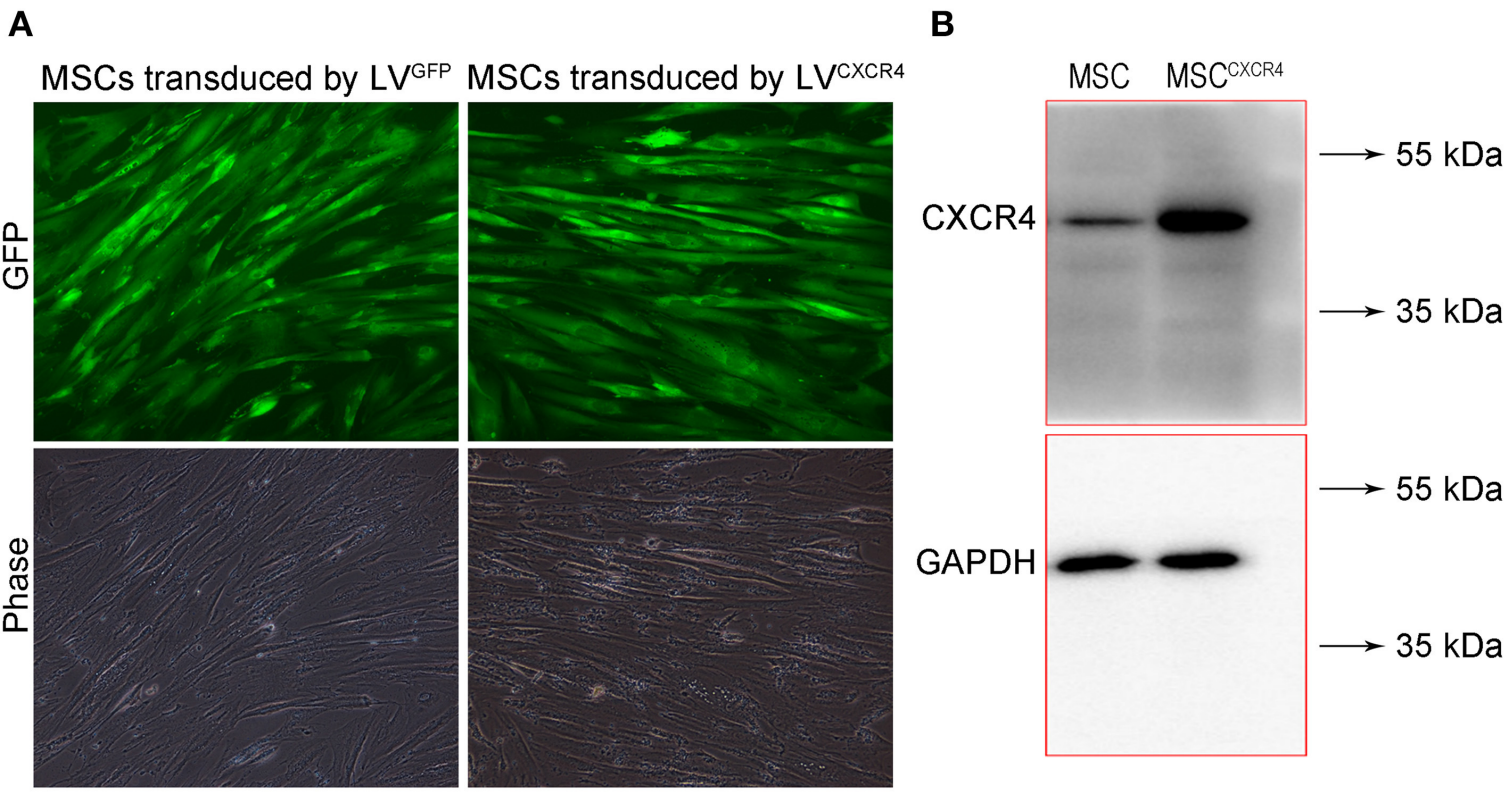
Characterization of MSC^CXCR4^ transduced by LV^CXCR4^. **(A)** Green fluorescence of MSC^CXCR4^ transduced by LV^CXCR4^ observed under fluorescent micrographs, with MSCs transduced by LV^GFP^ as the positive control group. **(B)** Expression of CXCR4 in MSCs and MSC^CXCR4^ identified by Western blotting. LV^CXCR4^, the lentivirus containing CXCR4 and GFP; LV^GFP^, the lentivirus containing GFP; MSC^CXCR4^, MSCs overexpressing CXCR4.

**Figure 2:**
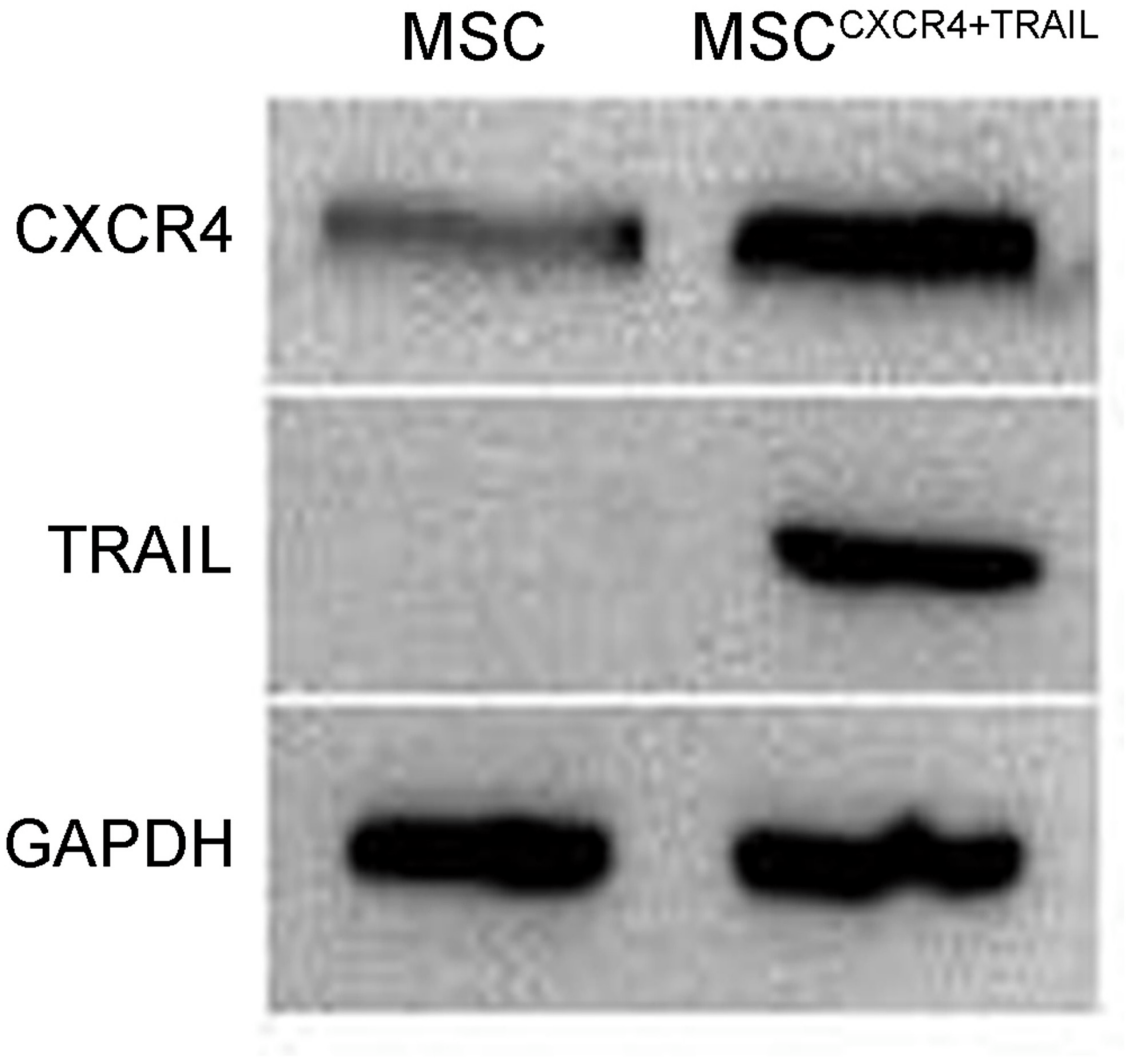
Expression of CXCR4 and TRAIL in MSCs and MSC^CXCR4+TRAIL^ identified by Western blotting. MSC^CXCR4+TRAIL^, MSCs overexpressing CXCR4 and TRAIL.

To evaluate CXCR4 expression, the fluorescence of MSC^CXCR4^ transduced by LV^CXCR4^ was observed under fluorescent micrographs, with MSC transduced by the lentivirus containing GFP (LV^GFP^) as the positive control group. Green fluorescence was observed in the MSC^CXCR4^ group, similar to that of the MSC group (Figure 1A). The result revealed that CXCR4 gene did not influence MSCs in terms of the MSC morphology and fluorescence intensity. Moreover, Western blots performed after the transduction by LV^CXCR4^ revealed that MSC^CXCR4^ exhibited a significantly higher level of CXCR4 than the MSCs (Figure 1B). This demonstrated that the lentiviral vector had transduced CXCR4 into the MSCs successfully. Based on the results, the evaluation of MSC^CXCR4+TRAIL^ was further carried out. The presence of CXCR4 and TRAIL in MSC^CXCR4+TRAIL^ after transduction by LV^CXCR4^ and LV^TRAIL^ was confirmed by Western blotting. Western blots revealed that MSC^CXCR4+TRAIL^ exhibited an apparent increase in CXCR4 level, and expression of TRAIL was also identified (Figure 2). This demonstrated that the lentiviral vector had transduced CXCR4 and TRAIL into MSCs successfully.

### Identification of Exo^CXCR4+TRAIL^

**Figure 3:**
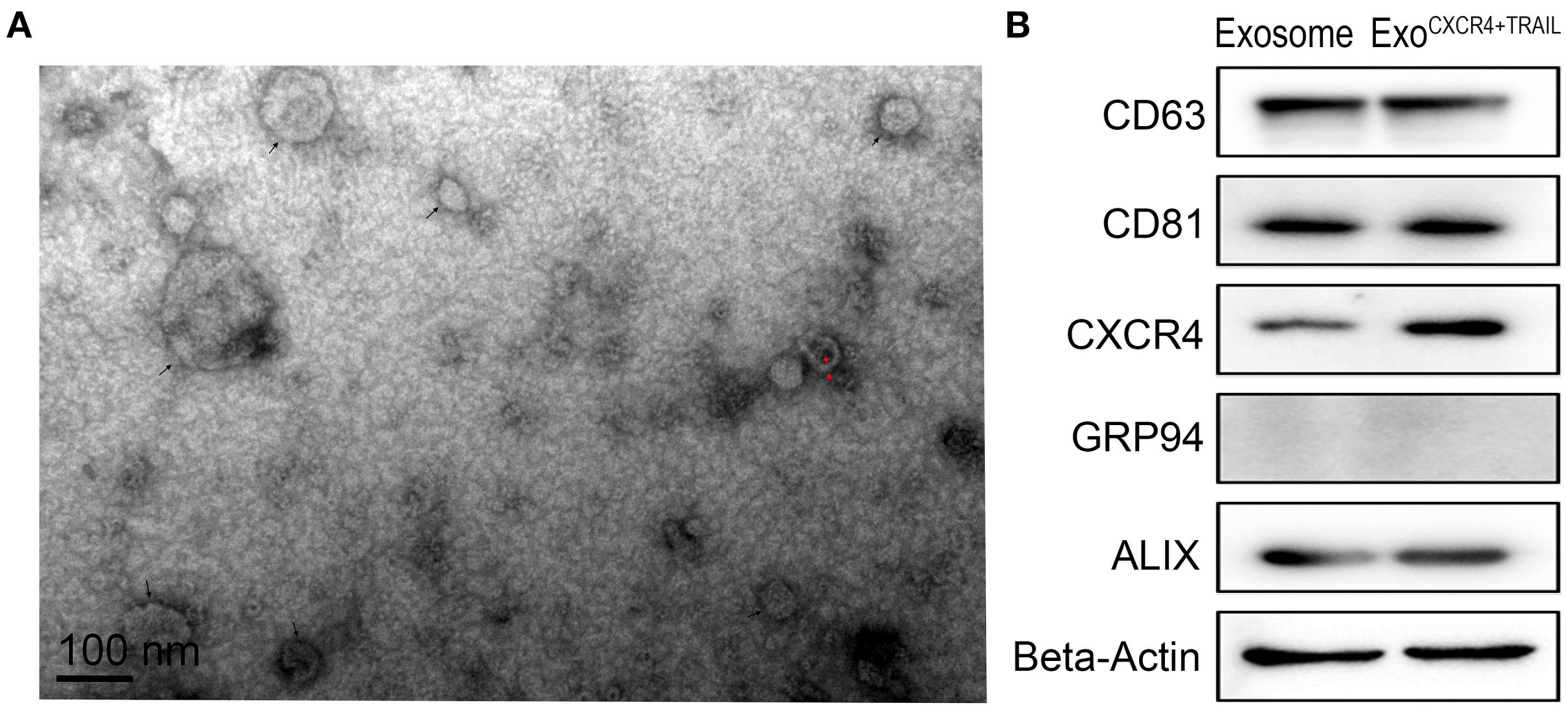
Characterization of Exo^CXCR4+TRAIL^. **(A)** Transmission electron micrograph of Exo^CXCR4+TRAIL^. Isolated exosomes are spherical (at the point of the black arrow) and membrane-encapsulated bodies (at the point of the red arrow) with a size of 40–90 nm. **(B)** Expression of exosomal markers in exosomes and Exo^CXCR4+TRAIL^ identified by Western blotting. Exo^CXCR4+TRAIL^, exosomes isolated from MSCs transduced by CXCR4 and TRAIL with lentiviral vector.

**Figure 4:**
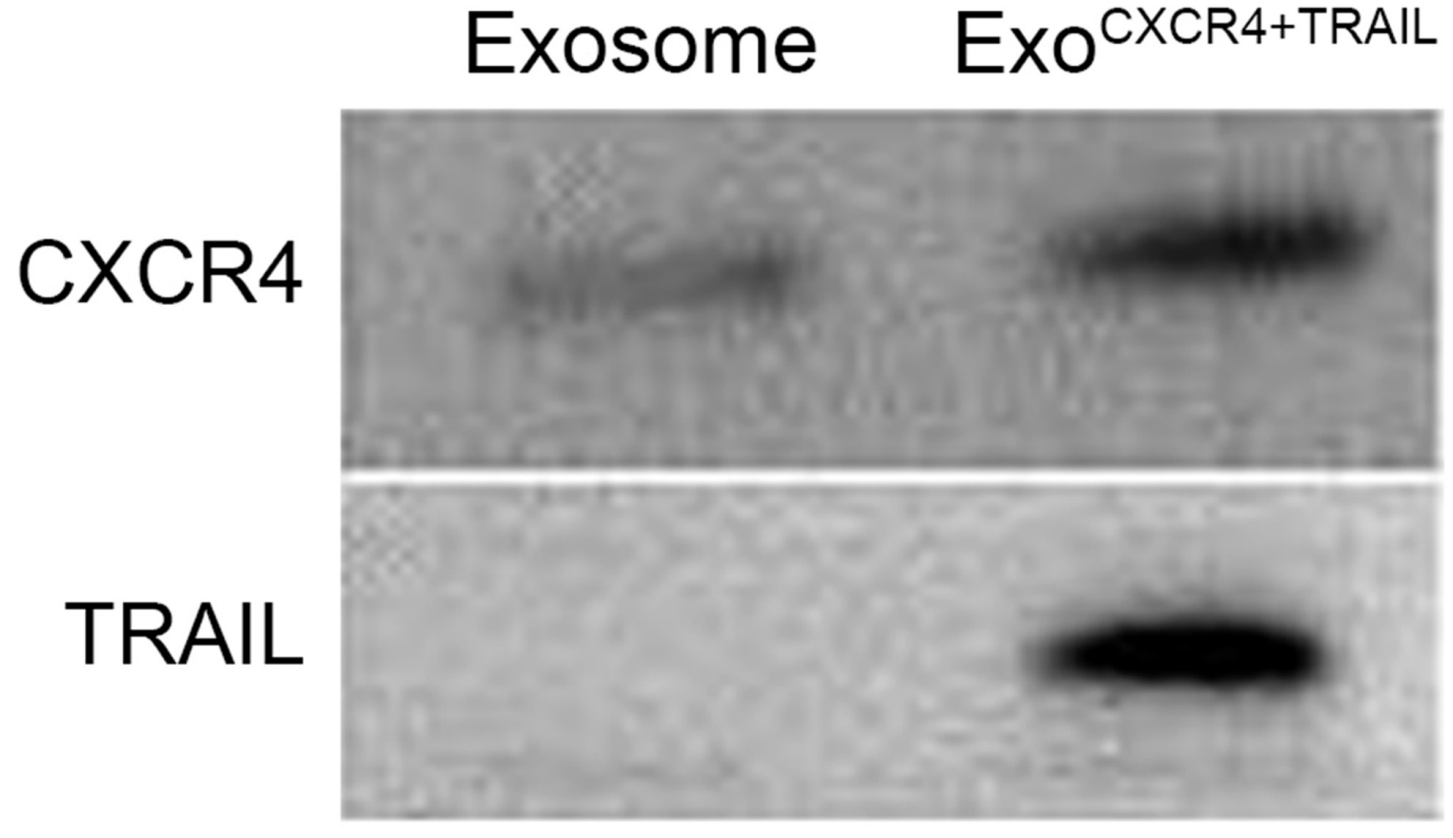
Expression of CXCR4 and TRAIL in exosomes and Exo^CXCR4+TRAIL^ identified by Western blotting. Exo^CXCR4+TRAIL^, exosomes isolated from MSC transduced by CXCR4 and TRAIL with lentiviral vector.

From the TEM results, Exo^CXCR4+TRAIL^ showed double-membrane and rounded structures (red arrow) with a size of 40–90 nm (Figure 3A), which is similar to previous descriptions (30, 31). Western blot analysis confirmed that the Exo^CXCR4+TRAIL^ fraction expressed CD63, CD81, and ALIX, all of these being well-established exosome markers (Figure 3B) (32). Furthermore, it was observed that Exo^CXCR4+TRAIL^ could be influenced by LV^CXCR4^ and LV^TRAIL^ transduction, as indicated by significant upregulation of the CXCR4 and TRAIL expression levels in comparison with exosome (Figures 3B, 4). The results revealed that CXCR4 and TRAIL were transduced into Exo^CXCR4+TRAIL^ without changing the main phenotypic expressions of the specific surface molecules of exosomes, which is an advantage in gene modification of exosomes, as reported previously (32).

### Anti-tumor Study of Carboplatin Combined With Exo^CXCR4+TRAIL^ in Brain Tumors

**Figure 5:**
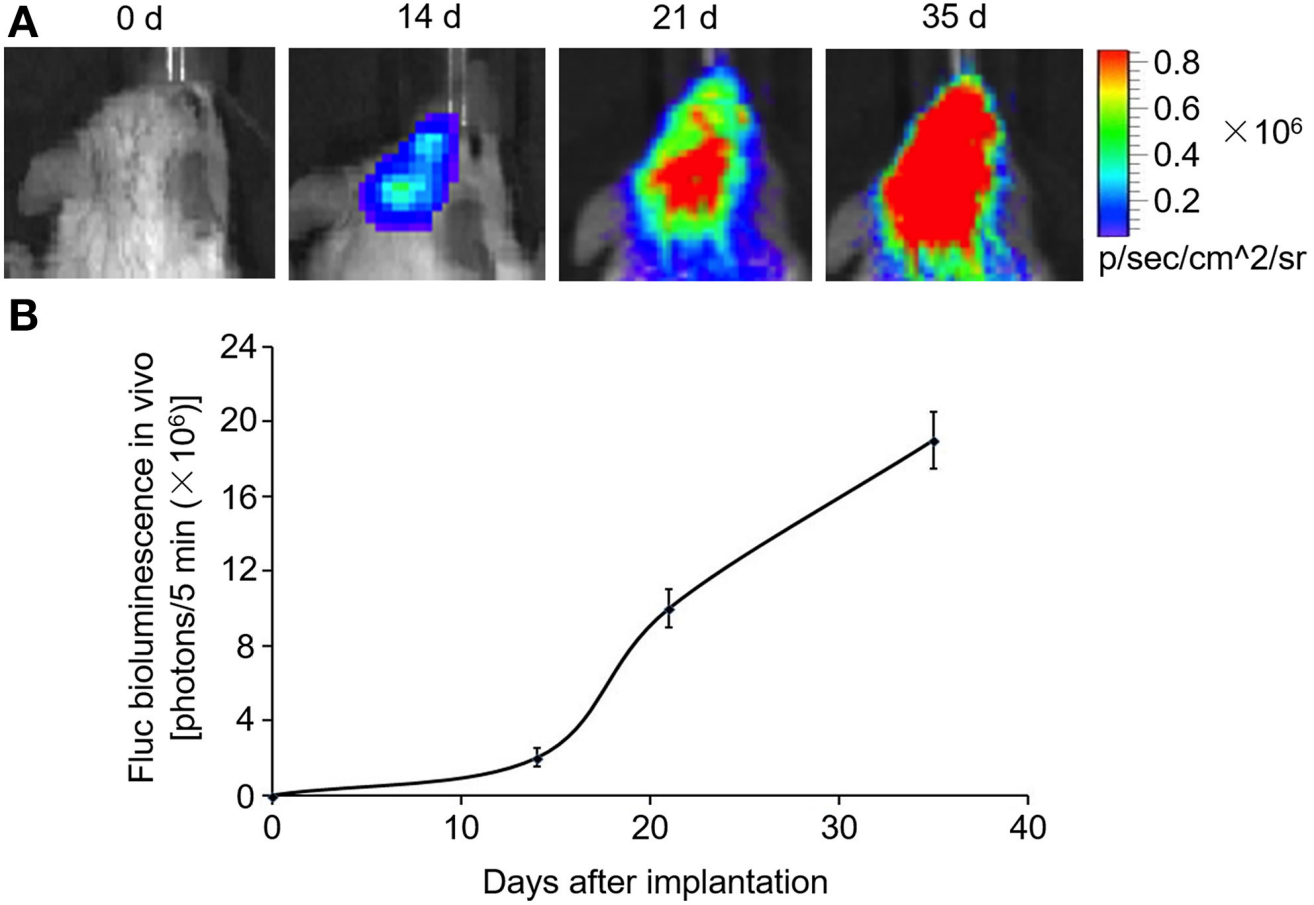
Construction of brain metastasis of a breast cancer model. MDA-MB-231Br-Luc cells (5 × 10^5^) implanted intracranially into SCID mice. **(A)** SCID mouse brains imaged with the *in vivo* imaging system on days 0, 14, 21, and 35 after cell implantation. **(B)** Fluorescence intensity in mice detected by quantitative analysis on days 0, 14, 21, and 35, respectively. All values expressed as mean ± SD; *n* = 6 for each group.

**Figure 6:**
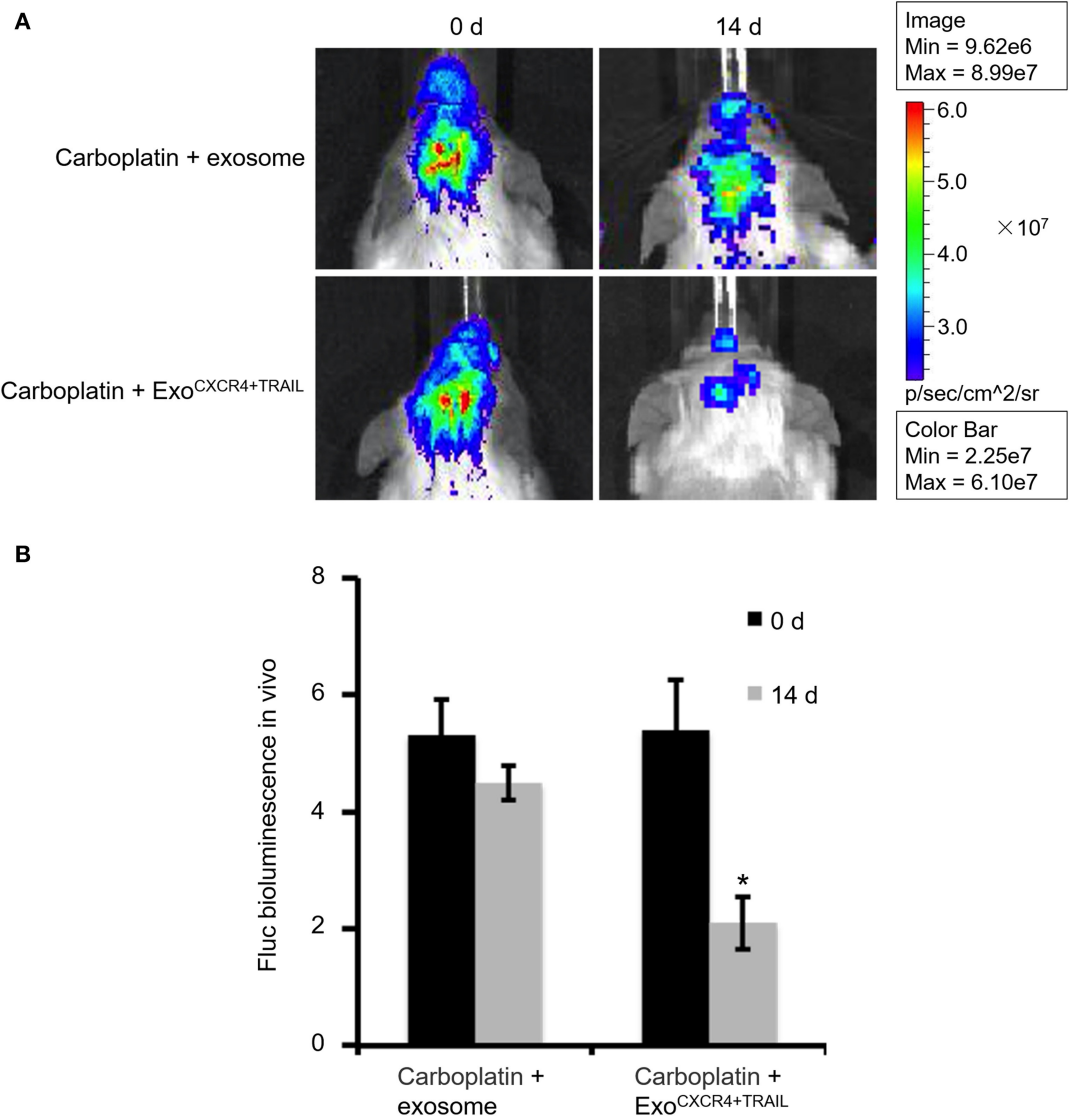
Fluorescence intensity in SCID mice administered with carboplatin (5 mg/kg) in combination with exosome (4 mg/kg) (Carboplatin + exosome group) and Exo^CXCR4+TRAIL^ (4 mg/kg) (Carboplatin + Exo^CXCR4+TRAIL^ group) on alternate days for 14 days after constructing the brain metastasis of breast cancer model **(A)** observed by the *in vivo* imaging system and **(B)** detected with quantitative analysis on days 0 and 14, respectively. All values expressed as mean ± SD. Exo^CXCR4+TRAIL^, exosomes isolated from MSCs transduced by CXCR4 and TRAIL with lentiviral vector. ^*^P < 0.05: “Carboplatin + Exo^CXCR4+TRAIL^” group vs. “Carboplatin + exosome” group; *n* = 6 for each group.

SCID mouse brains were imaged using the *in vivo* imaging system on days 0, 14, 21, and 35 after MDA-MB-231Br-Luc cells were implanted intracranially into the SCID mice (Figure 5A). The fluorescence intensity in mice was observed quantitatively on days 0, 14, 21, and 35, respectively (Figure 5B). Figure 5 shows that the bioluminescence value and area in the brain increased continuously with time. Moreover, at the beginning of the 14th day, the early stage of tumor growth in SCID mice brain was observed. Therefore, on the 14th day, after the brain metastasis of breast cancer model was established, the SCID mice were administered with carboplatin (5 mg/kg) in combination with Exo^CXCR4+TRAIL^ (4 mg/kg) (Carboplatin + Exo^CXCR4+TRAIL^ group) or exosome (4 mg/kg) (Carboplatin + exosome group) on alternate days for 14 days. The fluorescence intensity in both of the groups was observed by the *in vivo* imaging system (Figure 6A) and detected with quantitative analysis (Figure 6B) on day 0 and 14, respectively. Figure 6 shows that the signal of the tumor in the experimental group was decreased compared to the controls (*P* < 0.05), which indicates that Exo^CXCR4+TRAIL^ has the potential to enhance the antitumor effect of carboplatin.

## Discussion

MSCs have been focused upon because of their unique biological properties *in vivo*, which enable them to be used for the treatment of many pathological conditions (33). In cancer, MSCs play a crucial role in promoting tumor progression. They also contribute to tumor growth and progression in different cancer types (34), although their anti-tumor activities have also been reported (35). MSCs provide a framework for anchoring tumor cells in the form of tumor stroma, and then secrete factors that facilitate tumor growth (36). Moreover, under the influence of cytokines or chemokines, they can transdifferentiate into M2-type microphages, myeloid-derived suppressor cells, or M2-type macrophages in the tumor microenvironment (37–39). Furthermore, Nakamizo A. et al. and our previous study have proved that during the initial period of administration, the majority of MSCs were reported to be filtered by the lung and that only rare MSCs were integrated into the tumor (40, 41). This property could impact the benefit of MSCs when applied for the treatment of brain diseases.

Nowadays, endosome-derived exosomes, a kind of extracellular vesicles, have emerged as physiologically related and meaningful components of the MSC secretome (42). Exosomes regulate neurite outgrowth (43), promote angiogenesis (44), reduce myocardial ischemia/reperfusion injury (45), and repair acute kidney injury (46). Proteomic analysis reveals that MSC-derived exosomes have characteristic signaling molecules and critical surface markers of the MSCs (47). In addition to the advantages mentioned above, exosomes are also applied to deliver drugs across the BBB by decreasing MPS drug clearance (9, 10). Thus, it is reasonable to hypothesize that exosomes have gene therapy potential for brain diseases.

Based on the reports mentioned above, exosome derived from MSCs was applied as a tool for gene delivery. Therefore, gene modification of MSCs is an important part of the study. In order to explore the therapeutic potential of MSCs, genetically modified MSCs expressing different factors have been studied (48). In terms of safety and efficacy, lentiviral vectors may be the best tool for producing stable genetically modified MSCs. The lentiviral vectors have little influence on the biology of the transduced cells and can realize multiple modifications in their backbone. They are available on the market and in research groups and can be used to obtain constitutive or inducible transgene expression in MSCs (49). In other words, lentiviral vectors have turned out to be one of the highest-potential and most useful vehicles for MSC gene modification.

TRAIL is a member of the tumor necrosis factor ligand superfamily (50). It exerts minimal activity against most normal cell types (50, 51) but is highly expressed in several kinds of solid tumors (22). Cytotoxic chemotherapeutics can regulate the expression of TRAIL receptors (52, 53). Moreover, they can modify response to TRAIL receptor agonists by changing the levels of pro- and anti-apoptotic regulatory proteins (54, 55). Based on this consideration, a combination of cytotoxic chemotherapy with agents targeting TRAIL receptors showed the potential of synergistic proapoptotic effects. Additionally, CXCR4, a G-protein-coupled receptor, can activate several related downstream signaling pathways after stimulation. This is a key factor related to several processes, such as homing and endothelial cell migration (17, 18). Thus, after CXCR4 gene modification, the ability of exosomes from MSCs to home to disease sites should be enhanced.

Based on the above considerations, this study is the first to present the preparation of Exo^CXCR4+TRAIL^ derived from MSCs. Utilizing the ability of Exo^CXCR4+TRAIL^ to cross the BBB and its advantages of being an excellent gene vector, we investigated whether a combination of CXCR4, TRAIL, and carboplatin used in the clinic was effective to treat a brain metastasis of breast cancer model. From Figure 6, we could find that Exo^CXCR4+TRAIL^ has potential for use in the treatment of brain metastases of breast cancer, but further study is needed of its associated mechanisms, such as whether the vector can effectively cross the BBB and whether apoptosis occurs in the brain tumors. In addition, further evaluation of the safety of the vector in animals during the treatment is necessary. In future research, we will use carboplatin or only exosome as a single-treatment group to reveal the differences among the groups more comprehensively. In the meanwhile, a survival plot for the anti-tumor effect should be produced. Considering the problem that there are highly heterogeneous responses even in syngeneic mice, the images of more mice should also be investigated. Moreover, histological evaluation is a very important method for the further study of the relationship between the shrinkage of tumor on fluorescence imaging and tumor cell apoptosis. Besides, the uptake of Exo^CXCR4+TRAIL^ and its effect on MDA-MB-231Br cells will be investigated in the next step, which could help us provide comprehensive evidence to show that CXCR4/TRAIL-enriched exosomes exerted this role by targeting and apoptosis induction. Furthermore, tumor marker quantification under the influence of exosomes needs further study. Analysis of proteins and cytokines involved in the tumor response will be needed to conclude that the present gene therapy strategy is working.

## Conclusion

CXCR4/TRAIL-enriched exosomes were successfully obtained from MSCs overexpressing both CXCR4 and TRAIL, which were characterized by TEM and Western blotting. These exosomes exerted activity as a cooperative agent with carboplatin against brain metastasis of breast cancer *in vivo*. This novel application of CXCR4/TRAIL-enriched exosomes in improving the efficacy of chemotherapy highlights a new perspective for establishing a synergistic protocol with anticancer agents to treat brain disease. Furthermore, CXCR4-mediated targeting and TRAIL-mediated apoptosis induction in tumor cells suggests that Exo^CXCR4+TRAIL^ may be used as an optional therapeutic tool, potentially providing a novel approach for advancing the treatment of brain metastases of breast cancer with anticancer agents. In addition, it is important to determine the effect of Exo^CXCR4+TRAIL^ on BBB and in apoptosis induction in brain tumors. Furthermore, it is necessary to conduct a safety evaluation of the vector in animals during treatment.

## Data Availability Statement

The original contributions presented in the study are included in the article/supplementary material, further inquiries can be directed to the corresponding author/s.

## Ethics Statement

The animal study and experiments were reviewed and approved by the Animal Ethics Committee of Zhejiang University. All animal procedures were performed in accordance with Health Guidelines for the Care and Use of Laboratory Animals of Zhejiang University.

## Author Contributions

ML and GC: conceptualization and validation. YH: methodology, resources, writing—review and editing, and project administration. ML: software, formal analysis, data curation, writing—original draft preparation, and visualization. GC: investigation. YH and GC: supervision. YH and ML: funding acquisition. All authors have read and agreed to the published version of the manuscript.

## Conflict of Interest

The authors declare that the research was conducted in the absence of any commercial or financial relationships that could be construed as a potential conflict of interest.
